# Hamstring muscle architecture and myotonometer measurements in elite professional football players with a prior strained hamstring

**DOI:** 10.5114/biolsport.2023.112092

**Published:** 2022-01-03

**Authors:** F. Javier Núñez, Juan Carlos Martínez, Jan-Arie Overberg, Nacho Torreno, Luis Suarez-Arrones

**Affiliations:** 1 Universidad Pablo de Olavide, Physical Performance & Sports Research, Seville, Spain; 2 Performance and Health Department, FC Basel 1893, Basel, Switzerland

**Keywords:** Injury, Biceps femoris long head, Fascicle length, Angle pennation, MyotonPRO, Soccer

## Abstract

The purpose of this study was to compare the fascicle length, angle pennation and mechanical properties of the biceps femoris long head (BFlh) in dominant and non-dominant limbs in previously injured and uninjured professional football players. Fifteen professional football players were recruited to participate in this study. Seven players had suffered a BFlh injury during the previous season. Myotonometry mechanical properties were measured in the proximal, common tendon and distal BFlh using MyotonPRO, and angle pennation and fascicle length were also measured. We observed significantly higher distal BFlh frequency, stiffness, decrement, relaxation and creep than in the common tendon and proximal BFlh. The previously injured players showed significantly higher frequency and stiffness, and lower relaxation and creep in the dominant BFlh than did uninjured players. There were no significant differences between the fascicle length and angle pennation in previously injured and uninjured BFlh. Myotonometric measurement provides a quick and inexpensive way to check the properties of the BFlh in professional football players. Professional football players with previous BFlh injury showed higher intrinsic tension and a poorer capacity to deform than did players with no injury to the BFlh.

## INTRODUCTION

Hamstring strain injury (HSI) is the noncontact injury with the highest incidence in football (soccer) [1], accounting for 12–16% of all injuries. This injury has not shown signs of decrease over the last three decades [2]. The biceps femoris long head (BFlh) is the most commonly injured of the hamstring muscles [3], so there is considerable interest in intrinsic factors that predispose football players to BFlh injury [4]. BFlh injuries usually happen during the late swing phase in high-speed running or sprinting at long muscle lengths, and it has been suggested that relatively long muscle fibres can minimize the risk of injury by presenting less stress per sarcomere for a given muscle-tendon unit strain [5, 6]. Among many other well-documented risk factors [7], increased eccentric strength and alterations in muscle architecture (elongation of the BFlh fascicle) have been proposed as modifiable risk factors with a positive impact on hamstring injury prevention [8, 9]. However, the responses of both muscle architecture and strength to training are probably training status dependent; thus, extrapolating the research findings obtained in semi-professional or amateur football players to highly trained professional football players requires caution [10].

Fascicle length alterations and their impact on BFlh injuries have been investigated by a number of researchers [9, 11]. A previous article using B-mode ultrasound suggested that professional football players with shorter BFlh fascicles (i.e., < 10.5 cm) have a fourfold greater risk of HSI than players with longer fascicles [9]. Given that a previous HSI has been consistently shown to increase the risk of future injury [11], Timmins et al. showed, using B-mode ultrasound, that a previously injured BFlh consistently has shorter fascicles than the uninjured contralateral limb [12]. However, it is not clear whether these differences in fascicle length are due to the previous injury, or a result of decreased ability to activate the previously injured BFlh [13]. Therefore, analysis of the mechanical properties of the BFlh is needed to address this gap in knowledge. A number of ultrasound methods can be used to assess fascicle lengths. Extended field of view (EFOV) ultrasound has been shown to be superior to static ultrasound in assessing BFlh fascicle length by avoiding extrapolation errors [6]. Therefore, the results of previous studies using B-mode ultrasound should be interpreted with caution.

Examining the mechanical properties of a muscle is important in monitoring the state of the tissue, detection of possible fatigue and for assessing the efficacy of treatment/training interventions [14]. Therefore, reliable and sensitive methods for determining neuromuscular function as well as mechanical muscular properties are essential in professional football. Coaches at the elite football level are often concerned about the possible risks of invasive and maximal testing during intensive training periods, including fatigue, reduced trainability and/or possible future muscle injury [15]. Consequently, non-invasive measurement methods such as myotonometry are an alternative to monitor muscle mechanical properties objectively and regularly during the season. The MyotonPRO (Myoton AS, Estonia and Myoton Ltd London) is a handheld device that percutaneously applies a mechanical impulse with quick release to the muscle, resulting in muscle oscillations. The oscillations are then measured by an accelerometer and various parameters are calculated simultaneously. The myotonometric measurements obtained using this system have demonstrated good validity [16], optimal relative and absolute reliabilities [17], and high sensitivity to changes [18]. Several studies have shown the feasibility of using this device to monitor the mechanical properties of skeletal muscles in athletes [16].

The unilateral nature of football has been proposed as an important contributing factor to the aetiology of BFlh injury in football players [19]. Previous analyses of the fascicle length and muscle properties of BFlh involved comparisons of right vs left limbs [20] or uninjured vs contralateral injured legs [9, 11], regardless of which limb was dominant. In football the dominant limb is the leg usually used for kicking the ball [21]. Previous research showed that the dominant limb was associated with significant increases in strain magnitude in the BFlh, showing that the muscle strain was greater when kicking with the dominant than with the non-dominant limb [21]. The normal limb dominance characteristics were found to be affected in the presence of a previous BFlh injury [22]. On this basis, it is desirable to perform an analysis based on the dominance of the lower limb rather than its location (i.e. right vs. left limb).

Consequently, the purpose of this study was to compare the fascicle length, pennation angle and mechanical properties of the BFlh of injured vs uninjured legs in injured elite professional football players, and dominant vs non-dominant limbs in uninjured elite professional football players. It was hypothesized that fascicle length is shorter in the previously injured leg in comparison with the uninjured leg in elite professional football players. The properties of the BFlh can discriminate between the common tendon, distal and proximal regions, and between previously injured and uninjured legs in elite professional football players.

## MATERIALS AND METHODS

### Participants

Fifteen international-level elite professional football players were recruited to participate in this study (mean ± SD; age: 23.9 ± 3.6 years, height: 1.83 ± 0.09 m, and weight: 77 ± 10.4 kg). Eight players had no previous hamstring injuries and seven players had suffered a hamstring injury during the previous three seasons. Injured players obtained a diagnosis of functional disorder (14%), muscle strain injury I (43%) and muscle strain injury II (43%), and they returned to full activity following mild (4–7 days, 14%), moderate (8–28 days, 57%) and severe (> 28 days, 29%) injury [23]. The diagnosis of prior hamstring strain injury was made with MRI, ultrasound or a combination of both in most cases, in addition to the clinical assessment by the club doctor. The study was conducted according to the Declaration of Helsinki, and the protocol was fully approved by the ethics and research committee of the Virgen Macarena and Virgen del Rocío university hospitals (0398-N-17) before recruitment. After a detailed explanation of the aims, benefits, and risks involved in this investigation, all participants gave written informed consent.

### Procedure

### Ultrasound imaging

A specialist in sports ultrasonography, with 10 years of experience, performed a standardized ultrasound evaluation of the BFlh in the study participants, at the medical department of FC Basel (Switzerland). Ultrasound images were acquired with a GE-LogiQ S7 ultra-sound device (GE Healthcare, Milan, Italy) using a linear 50 mm transducer with an imaging depth field of 8 cm (ML6-15). Participants were instructed to rest in a prone position with the knees extended and the feet relaxed down the side of the bed for better comfort. They were also asked to remain relaxed during the image acquisition. In order to obtain panoramic images of the whole muscle, the EFOV ultrasound technique was used. An ultrasound identification was carried out over the muscle-tendon junctions (MTJs). While the transducer was oriented transversely to the long axis of the muscle, the probe was gradually moved distally or proximally to locate the distal or proximal MTJ respectively. The transducer was oriented longitudinally at the intersection of the superficial and deep aponeuroses, and a mark was drawn on the skin when the smallest muscle section was examined [24]. Once the MTJs were located, the best ultrasound imaging path for BFlh was marked along the horizontal plane by following its fascicles along the superficial compartment proximo-distally. To acquire the EFOV images, a constant pressure was applied to the transducer as it was moved slowly and continuously along the marked path from the proximal to the distal MTJ. The transducer orientation was continuously adjusted to keep it on the fascicle plane so that the fascicles would appear as a continuous and visible pattern, while the aponeuroses stayed parallel. To improve the acoustic contact and to keep the transducer pressure on the skin to a minimum, transmission gel was used for all scans. The region of interest was set in line with the site within the BFlh belly where both superficial and mid-belly aponeuroses were as parallel as possible, the muscle thickness (i.e. the distance between the superficial and midbelly aponeurosis) was the greatest, and the hyperechoic lines delineating the BFLH fascicles (i.e. perimysial membranes) were visible for the maximum length. Longitudinal and transverse scans were carried out to locate this region of interest. Repeated measures could then always be executed at the same site using a line purposely drawn on the skin surface corresponding to the midpoint of the region of interest and two markers drawn on both sides of the transducer so that it was in line with the midpoint of the sonogram field of view [25].

### Biceps femoris long head architecture analysis

An image-processing program (ImageJ 1.52p, National Institutes of Health, Bethesda, USA) was used to digitize fascicle length and pennation angle. With the goal of detecting suitable fascicles for analysis, the fascicle insertion point into the intermediate aponeurosis needed to be clearly evident and a reasonable portion of the fascicle (at least ˜25% of the total estimated length) needed to be perceivable within the ultrasound transducer’s field of view. Four fascicles in the same region of interest were considered for analysis. Fascicle length was the distance (mm) from the intermediate to the superficial aponeuroses, directly evaluated using the segmented line tool. This tool allowed fascicle and superficial aponeurosis curvature to be calculated. The angle between the drawn fascicle and the intermediate aponeuroses was measured as angle pennation (º) [6]. Fascicle length evaluation and angle pennation by the same assessor showed very high reliability *within the same day* (ICC 95% CI: 0.99 (0.99–1.00); CV (%): 1.0% (0.8–1.5%) and 0.99 (0.98–1.00); CV (%): 2.1% (1.6–3.1%), respectively).

### Myotonometry mechanical properties

The participants lay prone on a standard examination bed during measurement of the mechanical properties of BFlh with the Myoton-PRO (Myoton AS, Estonia and Myoton Ltd London). Oscillation frequency (F, Hz), logarithmic decrement (D, arbitrary units), dynamic stiffness (S, N/m), mechanical stress relaxation time (R, ms), and creep (C, Deborah number) were measured. Oscillation frequency describes the intrinsic tension of the muscle in the resting state with no voluntary contraction, indicating muscle tone. Dynamic stiffness indicates the resistance to contraction or an external force that deforms the muscle’s initial shape. Logarithmic decrement describes muscle elasticity in terms of the recovery of the muscle’s initial shape after contraction or removal of an external force. Mechanical stress relaxation time represents the time required for restoration of muscle shape after deformation due to voluntary contraction or removal of an external force. Creep signifies the gradual elongation of the muscle over time when constant tensile stress is applied. Measurement points, which included the common tendon and the proximal and distal position of each BFlh, were determined by ultrasound imaging as previously described. At each site, the mean value of two sets of five consecutive measurements was used for analysis. If the coefficient of variation was more than 3%, the measurement was repeated. The myotonometric measurements used in this system have shown good validity [16], optimal relative and absolute reliability [17] and high sensitivity to changes [18].

### Statistical analyses

Data are presented as mean ± SD. Before conducting statistical analyses, we tested the data distribution using the Shapiro-Wilk test of normality. We performed a two-way ANOVA to test all the possible interactions between measurement points and limbs, and the fascicle length and properties of BFLh. We also tested the main effects of those factors. Finally, we performed a post-hoc analysis for pairwise comparisons with a Bonferroni adjustment. We set the significance level at p < 0.05. For these the analyses, we used JASP statistical software (JASP Team, 2020, v.0.12.0) for Windows. The effect sizes (ES, 95% confidence limit [26]) of the selected variables were calculated using the pooled pre-training SDs. The threshold values for the Cohen ES statistics were > 0.2 (small), > 0.6 (moderate), and > 1.2 (large) [27]. The chance that any difference was better/ greater (i.e., greater than the smallest worthwhile change, or SWC [0.2 multiplied by the between-subject standard deviation based on the Cohen’s d principle, ES]), similar or worse/smaller than that of the other group was subsequently calculated. The probabilities of beneficial/better or detrimental/poorer effects were qualitatively assessed as follows [27]: < 1%, most likely not; > 1–5%, very unlikely; > 5–25%, unlikely; > 25–75, possible; > 75–95%, likely; > 95–99%, very likely; and > 99%, most likely. If there was a chance that the true value was > 25% beneficial and > 0.5% harmful, then the clinical effect was considered to be unclear [27]. However, the clinical inference was declared to be beneficial when the odds ratio of benefit/harm was > 66 [27].

## RESULTS

Descriptive data for BFlh mechanical properties in different measurement regions, fascicle length and pennation angle in injured players (IP: injured limb (IPI) and uninjured limb (IPU)) and uninjured players (IU: dominant limb (UPD) and non-dominant limb (UPND)) are shown in Tables 1 and 2 respectively. No significant time × group interaction was observed between variables. A significant effect was observed between different myometric regions of BFlh measured and frequency (η²_p_ = 0.306; p < 0.001), stiffness (η²_p_ = 0.245; p < 0.001), decrement (η²_p_ = 0.108; p = 0.021), relaxation (η²_p_ = 0.162; p < 0.001), and creep (η²_p_ = 0.220; p < 0.001). The elite professional football players showed significantly higher distal BFlh frequency, stiffness, decrement, relaxation, and creep than in the common tendon and proximal regions (see Table 1). A significant effect was observed between the injured players’ limbs (i.e. IPI and IPU), the uninjured players’ limbs (i.e. UPD and UPND) and frequency (η²_p_ = 0.160; p < 0.001), stiffness (η²_p_ = 0.145; p < 0.001), relaxation (η²_p_ = 0.140; p < 0.001), and creep (η²_p_ = 0.140; p < 0.001). The IPI and IPU showed significantly higher frequency (p = 0.004 and p = 0.05 respectively) and stiffness (p = 0.004 and p = 0.005 respectively) and significantly lower relaxation (p = 0.003 and p = 0.044 respectively) and creep (p = 0.002 and p = 0.05 respectively) than UPD. The IPI showed significantly higher frequency (p = 0.005) and stiffness (p = 0.002) and significantly lower relaxation (p = 0.013) and creep (p = 0.008) than UPND (see Table 2). There were no differences between IPI and IPU, or between UPD and UPND.

**TABLE 1:** Descriptive data of myotonometry mechanical properties for different measurement points of biceps femoris long head (BFLH). Data are Mean ± SD.

	Different measurement points of BFLH			Common Tendon vs. Dixtal			Proximal vs. Dixtal		
	Proximal (n = 30)	Common Tendon (n = 30)	Dixtal (n = 30)	ES (95%CI)	Probabilities	p value	ES(95%CI)	Probabilities	p value
Frecuency (Hz)	15.54 ± 1.75	15.82 ± 1.75	18.17 ± 1.5	1.28 ± 0.38	100/0/0	< 0.001	1.51 ± 0.38	100 / 0 / 0	< 0.001
Stiffness (N/m)	276 ± 49.6	283.5 ± 46.1	337 ± 33.9	1.09 ± 0.36	100/0/0	< 0.001	1.2 ± 0.35	100 / 0 / 0	< 0.001
Decrement (au)	1.365 ± 0.27	1.381 ± 0.26	1.219 ± 0.08	0.57 ± 0.32	0 / 0 / 100	0.035	0.45 ± 0.33	1 / 0 / 99	0.042
Relaxation (ms)	19.51 ± 3.04	18.87 ± 2.63	16.01 ± 1.51	1.14 ± 0.37	0 / 0 / 100	< 0.001	1.26 ± 0.36	0 / 0 / 100	< 0.001
Creep (dn)	1.2 ± 0.6	1.164 ± 0.14	1.01 ± 0.9	1.14 ± 0.37	0 / 0 / 100	< 0.001	1.28 ± 0.37	0 / 0 / 100	< 0.001

au: arbitrary units; dn: Deborah number

**TABLE 2:** Descriptive data for biceps femoris long head (BFLH) myotonometry mechanical properties, angle of penneation and length fascile, and comparation between limbs of injured players and uninjured players. Data are Mean ± SD

	Previously Injured Players (IP)		Uninjured Players (UP)		IPI vs. UPD		
	Injured Limb (IPI; n = 7)	Uninjured Limb (IPU; n = 7)	Dominant Limb (UPD; n = 8)	Non-Dominant Limb (UPND; n = 8)	ES(95%CI)	Probabilities	p value
Frecuency (Hz)	17.63 ± 1.68	17.34 ± 1.85	15.68 ± 1.81*^†^	16.15 ± 2.14*	1.01 ± 0.51	100 / 0/ 0	0.031
Stiffness (N/m)	331 ± 37.57	317.2 ± 42.67	279.5 ± 48.89*^†^	288.3 ± 42.67*	0.95 ± 0.44	100 / 0/ 0	0.02
Decrement (au)	1.367 ± 0.222	1.351 ± 0.213	1.298 ± 0.234	1.301 ± 0.247	0.30 ± 0.53	83 / 0 / 17	1
Relaxation (ms)	16.38 ± 1.75	17.08 ± 2.33	19.33 ± 3.035*^†^	18.59 ± 3.007*	0.98 ± 0.47	0 / 0 / 100	0.017
Creep (dn)	1.032 ± 0.1	1.066 ± 0.130	1.194 ± 0.165*^†^	1.146 ± 0.159*	0.98 ± 0.48	0 / 0 / 100	0.015
Penneation Angle (Degrees)	16.58 ± 5.66	17.10 ± 6.56	16.21 ± 4.67	16.86 ± 5.37	0.06 ± 0.82	55/0/45	1
Length Fascile (mm)	8.49 ± 0.94	8.56 ± 0.95	9.356 ± 1.257	9.311 ± 1.295	0.64 ± 0.77	8 / 0 / 92	0.933

* Significant differences with IPI;

† Significant differences with IPU; au: arbitrary units; dn: Deborah number

## DISCUSSION

The purpose of this study was to compare the fascicle length, pen-nation angle and mechanical properties of the BFlh of injured vs. uninjured legs in injured elite football players, and dominant vs. non-dominant limbs in uninjured elite football players. The main findings of this study were: a) the properties of the BFlh were different in the distal region in comparison with the proximal region and common tendon; b) frequency, stiffness, relaxation, and creep properties of BFlh were different in previously injured players vs. uninjured players; and c) there were no differences among injured players in fascicle length and pennation angle of BFlh between previously injured and uninjured legs, or between the dominant and non-dominant limbs in uninjured players.

The present study is the first to explore the mechanical properties of the BFlh using myotonometric measurements in elite professional football players. In football, winning ball possession, passing a defending player or gaining a position to score a goal are determined by the player’s ability to accelerate [28]. This acceleration is strongly determined by the horizontal ground reaction force and therefore by the football player’s ability to activate their hamstring muscles just before ground contact, which has the greatest capacity to produce eccentric knee flexor peak torque [29]. It has been reported that football players returning to play after rehabilitation from a hamstring injury can display a decrease in horizontal force production [30]. Although some research showed that injured athletes may experience apprehension about experiencing pain when producing a high level of force [31], our results agree with these findings since the IPI and IPU BFlh in elite professional football players may have less potential to produce eccentric peak torque, as extrapolated from myotometer data (i.e. higher F and S, and lower C) [18]. From our experimental design, we could not determine whether the decrease in horizontal force production was linked to knee flexion or hip extension of the BFlh. However, our results indicated that in elite professional football players, the distal region of the BFlh showed a poor capacity to produce eccentric force (i.e. higher F and S, and lower C) compared to the common tendon area or proximal region of the BFlh (see Table 1). Based on the knowledge that a more compliant musculotendinous system has a greater capacity to elongate, enabling external forces to be absorbed over a greater distance and time, thereby creating a cushioning effect [32], our myotonometric values showed higher intrinsic tension in the muscle in the resting state (i.e. F), higher resistance to external force that deforms the muscle’s initial shape (i.e. S) and a significant decline in the ability to provide a gradual elongation of the muscle over time when constant tensile stress is applied (i.e. C), in IPI compared to UPD and UPND and IPU compared to UPD. There are no myotonometric values of BFlh for this cohort (elite professional football players); therefore we cannot compare our data with other studies in professional football. However, our results are in line with a prospective study with elite netball players that found higher stiffness (i.e. F) in the soleus and Achilles tendon of injured compared with uninjured athletes [33]. In our case, there were no differences between injured and uninjured limbs in the injured players, or between dominant and non-dominant limbs in the uninjured players. While it is evident that passive muscle stiffness measured using myotonometry is sensitive to changes induced by eccentric exercises [34], further research is needed to track BFlh injuries in football players over time, and to analyse whether the stiffness is a contributing factor in return to play.

It is known that football training and competition induce greater increases in concentric than eccentric strength in the BFlh [35], favouring shortening of the fascicles of this musculature [36, 37]. The fascicle lengths of elite professional football players in our study (˜9 cm) were slightly longer than those observed in under-19 football players (8.5 cm) [8], although shorter than those observed in Australian professional football players (10.63 cm.) [9], senior football players (10.12 cm) [20] and young professional football players (10.55 cm) [4], before subjecting them to specific training programmes to increase the fascicle length. The younger age of the football players in the study by Lacome et al. [8] could also explain the slight differences in comparison with our results (both studies used EFOV), as muscles and fascicles generally tend to lengthen with growth [38]. Comparison with the other studies would not be appropriate because, as Franchi et al. [6] showed, the linear extrapolation of visible fascicles from a single ultrasound image results in an overestimation of BFlh when compared with EFOV scans, which could explain the differences between studies.

Previous research suggests that professional football players with shorter fascicles (fewer in-series sarcomeres) may be more susceptible to overstretching and damage caused by powerful eccentric contractions, such as those performed during the terminal swing phase of high-speed running [9]. In addition, Timmins et al. [12] observed that previously injured limbs had shorter BFlh fascicles than the contralateral uninjured limbs and the two-limb-average of the uninjured players when assessed at rest. In contrast to this and using EFOV ultrasound scans, our results with elite professional football players showed that there were no differences in fascicle length between the previously injured limb in comparison with the uninjured limb. The method by which the return to play process after injury is performed will have a substantial impact on the subsequent state of the BFlh muscle tissue. Despite the nonuniform nature of BFlh architecture [39], a possible explanation for this fact could be that the elite football players analysed in our study were fully recovered from their hamstring injury with no repercussions or sequelae, and were training and competing weekly in elite professional football.

This study has some limitations associated with the ultrasound methodology to infer muscle fascicle lengths [20, 25, 40]. However, the method used in this study, manual linear extrapolation, has recently been recommended due to its lower fascicle estimation and greater accuracy in comparison with the trigonometric equation methods used in other studies [6].

## CONCLUSIONS

We describe, for the first time, reference values for BFlh fascicle length with EFOV scans, pennation angle and myotonometric values in previously injured vs uninjured limbs and in dominant vs non-dominant legs in elite professional football players. The properties of the BFlh were different in the distal region in comparison with the proximal region and the common tendon area. The previously injured limb showed higher intrinsic tension in the muscle in the resting state, higher resistance to external force that deforms the muscle’s initial shape, and a significantly reduced ability to provide a gradual elongation of the muscle over time when constant tensile stress was applied, but a similar muscle architecture. The BFlh fascicle lengths and pennation angle in elite professional football players showed no differences in previously BFlh injured vs uninjured legs or in dominant vs non-dominant limbs in uninjured players.
